# Histone deacetylase (HDAC) 11 inhibits matrix metalloproteinase (MMP) 3 expression to suppress colorectal cancer metastasis

**DOI:** 10.7150/jca.66914

**Published:** 2022-03-28

**Authors:** Yuqing Wen, Xuemei Zhang, Xiayu Li, Li Tian, Shourong Shen, Jian Ma, Feiyan Ai

**Affiliations:** 1 Department of Gastroenterology, the Third Xiangya Hospital, Central South University, Hunan Key Laboratory of Nonresolving Inflammation and Cancer, Changsha, Hunan, China.; 2 Cancer Research Institute and School of Basic Medical Science, Central South University, Changsha, China.; 3 Key Laboratory of Carcinogenesis and Cancer Invasion of the Chinese Ministry of Education, NHC Key Laboratory of Carcinogenesis, Changsha, China.; 4 Department of Pathology, Affiliated Hospital of Guilin Medical University, Guilin, Guangxi, China.

**Keywords:** HDAC11, colorectal cancer, invasion and metastasis, MMP3, H3K9ac

## Abstract

Emerging evidence has implicated invasion and metastasis are the major common reason of treatment failure and the leading cause of death in colorectal cancer (CRC). Many members of the HDAC family have been reported to be key factors in the genesis and progression of cancer. Until now, few research focused on the actual expression patterns of HDAC11 in most malignancies. In the current study, we found that the expression of HDAC11 is decreased in mouse colitis tissues and colitis-associated cancer (CAC) tissue compared with normal colon tissue. Clinically HDAC11 expression is significantly lower in colorectal cancer tissues of patients and correlated with lymph node metastasis. Additionally, HDAC11 is downregulated in the relative high metastatic potential colorectal cancer cells. We also found HDAC11 inhibits the migration and invasion of colorectal cancer cell by downregulating Mmp3 expression. At the molecular level, the expression of HDAC11 inversely correlated with the level of histone H3K9 and H3K14 acetylation. In addition, analysis of chromatin-protein association by ChIP-qPCR demonstrated that the level of H3K9 acetylation correlated with the upregulation of Mmp3. Through a better understanding of this previously unknown role of HDAC11 in migration and invasion of colorectal cancer, HDAC11 may become a novel candidate for developing rational therapeutic strategies.

## Introduction

Colorectal cancer (CRC) is one of the most common malignant tumor of digestive tract. In 2018, colorectal cancer remains the most commonly diagnosed gastrointestinal cancer, representing 1.8 million cases and 881,000 deaths globally, and constituting one in ten cancer cases and deaths 1. Colorectal cancer patients are getting younger and it is the third cause of death in malignant tumor death 2. Despite of early screening and development of new chemotherapeutic strategies, CRC still causes more than 600,000 cancer related deaths each year according to the World Health Organization statistics 3.To investigate its reason, the occurrence of invasion and distant metastasis are the crucial events that affect the prognosis and treatment effect of colorectal cancer. Therefore, dissecting the mechanism of colorectal cancer metastasis is considered to be a promising strategy for reducing its mortality.

Histone deacetylases (HDACs) downregulate the transcription of genes by removing the acetyl groups and increasing chromatin compaction. The HDAC family contains 18 proteins, which are divided into 4 classes based on their structure and homology. Class I HDACs include HDAC1/2/3/8; class II HDACs consist of HDAC4/5/6/7/9/10; class IV HDACs contain HDAC11 and class III are classified as sirtuins (SIRT 1-7). Aberrant expression of HDACs is often associated with carcinogenesis and cancer clinical prognosis 4, 5. Recent studies have suggested that HDAC5 and HDAC9 are significantly upregulated in high-risk medulloblastoma in comparison with low-risk medulloblastoma and be associated with poor survival 6.It also has been reported that HDAC2 expression is significantly associated with CRC progression 7. HDAC11, the sole member of HDAC class IV, was identified in 2002 as a new member of the zinc-dependent HDAC family 8. Although recent experiments indicated that it is expressed by antigen-presenting cells and is involved in regulating the immune response 9, regulates oligodendrocyte-specific gene expression and oligodendrocyte development 10, and regulates myeloid derived suppressor cell expansion and function 11, the expression level and roles of HDAC11 in colorectal cancer are largely unknown.

MMPs are a family of zinc-dependent endopeptidases comprised of gelatinases, collagenases, stromelysins, matrilysins and membrane-type MMPs. They can break down the basement membrane allowing cancer cells to metastasize and thus contribute to the invasive and metastatic potential of tumors. MMP3 is a widely studied matrix metalloproteinase that can regulate cell migration and invasion of cancer cells. Multiple studies uncovered an essential role for MMP3 in multiple stages of cancer and may serve as early diagnostic biomarker in bladder cancer 12, 13. The regulation mechanism of MMP expression or activity has been studied intensively, including transcriptional regulation, post-transcriptional regulation, the coagulation system and MMP activation 14. However, how HDACs regulating expression of MMP3 remains unclear.

In this study, we show that HDAC11 is downregulated in colitis-associated cancer (CAC) mouse model and human patients with colorectal cancer and inversely related to lymph node metastasis. Moreover, our results indicate that the expression of HDAC11 inversely correlated with the level of histone H3K9 and H3K14 acetylation. HDAC11 can down-regulate Mmp3 expression by reducing the level of histone H3K9 acetylation at the promoter of Mmp3. Additionally, we find a novel mechanism that HDAC11 inhibits colorectal cancer cell migration and invasion by down-regulating Mmp3 expression *in vitro*. Our studies identify a role for HDAC11 first as a suppressor of colorectal cancer metastasis.

## Materials and methods

### Cell lines and culture

The murine colon cancer cell line CT26.WT, murine macrophages (RAW264.7), and human colon cancer cell lines (SW480, HT-29, SW620, HCT116) were acquired from the Cancer Research Institute of the Central South University (Hunan, China). They were cultured in RPMI1640 medium supplemented with 10 % fetal bovine serum and cell culture conditions is 37 °C in a humidified incubator with 5% CO_2_.

### Plasmids construction and gene transfection

The full-length CDS of murine HDAC11 (NM_144919) or Mmp3 (NM_010809) was amplified by a PCR with murine colorectal tissue cDNA as template. The primers used were 5'-ATCGATATCATGCCTCACGCAACAC-3', 5'-TATGGATCCTCAAGGCACAGCACAG-3' and 5'-ATCGATATCATGAAAATGAAGGGTC-3', 5'-TATGGATCCTTAACAATTAAACCA-3'. The PCR cycles were as follows: 95 °C for 5 min followed by 30 cycles of 95 °C for 30 s, 56 °C for 30 s, and followed by a final extension at 72 °C for 2 min. After that, the PCR fragment was purified and was subcloned into the mammalian expression vector pIRES (Clontech, CA). The vectors were named pIRESneo3-HDAC11and pIRESneo3-Mmp3 for gene transfection. Plasmids were transfected into CRC cells using Lipofectamine 2000 (Invitrogen, Carlsbad, CA, USA).

### RNA interference

siRNA recognizing HDAC11 was synthesized from RiboBio (Guangzhou, China). The sequence of* Hdac11* targeted by the siRNA is as follows: GGTGATCAACTTCCTGAAA. For siRNA transfection, CRC cells were grown and transfected using HiPerFect Transfection Reagent (Qiagen, Valencia, CA, USA) following the manufacturer's protocol.

### Quantitative real-time PCR

Total RNA from CRC cells and tissues was extracted using Trizol (Invitrogen, CA). cDNA was synthesized from 2 μg of total RNA using a reverse transcription kit (Fermentas, Glen Burnie, MD, USA). The GAPDH gene was used as an internal control. Expression of the mRNAs was evaluated using qRT-PCR and a SYBR premix Ex Taq II kit in accordance with the standard protocol. Expression of each gene was quantified by measuring cycle threshold (Ct) values and normalized to GAPDH or GAPDH mRNA. The qRT-PCR primers used were human HDAC11 (forward, 5'-GCTATCTTAATGAGCTCAAGTGGT-3', reverse, 5'-TGTCCGCATAGGCACAGAAG-3', human GAPDH (forward, 5'-AACGGATTTGGTCGTATTGG-3', reverse, 5'-TTGATTTTGGAGGGATCTCG-3'), mouse HDAC11 (forward, 5'-CCATCCTCATGGTGACCTCG-3', reverse, 5'-TCAAGGCACAGCACAGGAAA-3'), mouse Mmp3 (forward, 5'-AGACTTGTCCCGTTTCCAT-3', reverse, 5'-GGTGCTGACTGCATCAAAGA-3') and mouse GAPDH (forward, 5'-TCTGACGTGCCGCCTGGAGA-3'), reverse, (5'-CAGCCCCGGCATCGAAGGTG-3').

### Western blot

Total cellular protein was extracted using a lysis buffer with protease inhibitor mixture (Roche, Rotkreuz, Switzerland). Equal protein samples (50μg) were separated by SDS-PAGE (Bio-Rad, CA) and then transferred to PVDF membranes (Millipore, MA, USA). Membranes were then probed with primary antibodies followed by secondary antibody. The signal detection was finally performed using an ECL detection system (Millipore, Billerica, MA, USA). Antibody against Mmp3 was purchased from Abcam (Cambridge, MA, USA). Antibody against HDAC11 was purchased from Santa Cruz Biotechnology (Santa Cruz, CA, USA). Antibody against GAPDH, anti-acetylated histone H3-K9 and H3-K14 antibodies were purchased from Millipore (Billerica, MA, USA).

### Tissue microarray

Tissue collection and the construction of tissue microarrays (TMA) have been described previously 15. Briefly, the TMA contained 336 dot matrices, including 103 cases of colorectal cancer, 103 cases of adjacent noncancerous mucosa (taken from 0.5-1.0 cm adjacent to primary tumor), 103 cases of distant morphological normal mucosa (>10 cm away from the primary tumor), and 27 cases of lymph node metastatic tumor tissues. All samples were H&E stained and examined by two senior pathologists to confirm the histopathologic state, including TNM stage and metastasis. Eighty-eight cases were well or moderately differentiated, and fifteen cases were poorly differentiated. The number of patients in stage I, stage II, stage III and stage IV is 3, 45, 30 and 25, according to the 7th edition of the AJCC TNM staging manual for CRC 16. Fifty-five patients had lymph node metastasis, and 48 had no lymph node metastasis. All procedures were approved by the Xiangya Hospital Ethics Committee of the Central South University and were performed after obtaining the patient's informed consent.

### Colitis-associated cancermouse models

The AOM/DSS-induced colitis-associated cancer mouse model was created as described by Tang *et al* 15. Animal procedures were performed in accordance with institutional guidelines.

### Native chromatin immunoprecipitation

The native chromatin immunoprecipitation (NChIP) assay was performed as described previously 17. In brief, the mouse colitis and normal tissue (from the above AOM-DSS-induced colitis mice model) were homogenized, micrococcal nuclease (MNase) digestion was carried out with a working concentration of 5U/mL at 37 °C for 10 min. Then the chromatin fragments were immunoprecipitated with antibodies specific to acetylated histone H3-K9 (Millipore), acetylated histone H3-K14 (Millipore) or control rabbit IgG (Millipore) at 4 °C overnight. After DNAs were dissociated from immunoprecipitated chromatin, the amounts of the specific to DNA fragments were quantified by real-time PCR.

Real-time PCR amplification was carried out with Power SYBR Green PCR Master Mix according to manufacturer's instructions (TaKaRa, Otsu, Japan). Primer sequences were: 5'-GTTGGGCTTAAGAAGGTGGA-3' (forward), 5'-GTGCTCATCCTACCCATTGC-3' (reverse). Data are presented as 'Relative Occupancy' by the equation 2^[ΔCt (IgG-Input) - ΔCt (Target-Input)]^.

### Immunohistochemistry

Immunohistochemistry (IHC) staining analysis for HDAC11 and MMP3 was carried out as previously described 15. Immunostained slides were observed under a microscope and staining intensity was scored as 0, negative; 1, weak; 2, intermediate; and 3, strong.

Staining extent according to the percentage of positive-stained cells was scored as follows: 0, <5%; 1, 5-25%; 2, 26-50%; 3, 51-75%; and 4, >75%. After that, the summation of the staining intensity and extent scores form a final score of each case: low expression (0-2 scores) and high expression (3-7 scores).

### Wound-healing assay

CT26.WT cells (1×10^6^) were transfected with DNA vectors and then grown in a 6-well plate. After the cell monolayer had reached 90% confluency, a wound was made with a 10-μl pipette tip. Cells were then cultured in medium with 1% serum, and images of the wound area were taken using a microscope (Nikon) at 0 and 24 h.

### Matrigel invasion Assay

Transfected cells were seeded in the chamber of 8-μm pores (Corning, New York, NY, USA) coated with Matrigel Matrix (BD Biosciences, San Diego, CA, USA). Normal growth medium was placed in the 24-well plate and the chamber was placed into it. The cells were then allowed to migrate for 24h at 37 °C. The invasive cells were fixed with 10% methanol for 15 min. Then the invasive cells were examined by crystal violet staining and the stained cells were counted under a microscope. To minimize bias, at least five and only selected fields with 100× magnification were counted, and the various counts were averaged.

### Statistical analysis

The significance of differences between groups was assessed using the Student's *t* test in SPSS 17.0 and GraphPad Prism 5. A p-value of <0.05 was considered to be statistically significant.

## Results

### Hdac11 is downregulated and Mmp3 is upregulated in chemically-induced CAC mouse model

**Figure 1:**
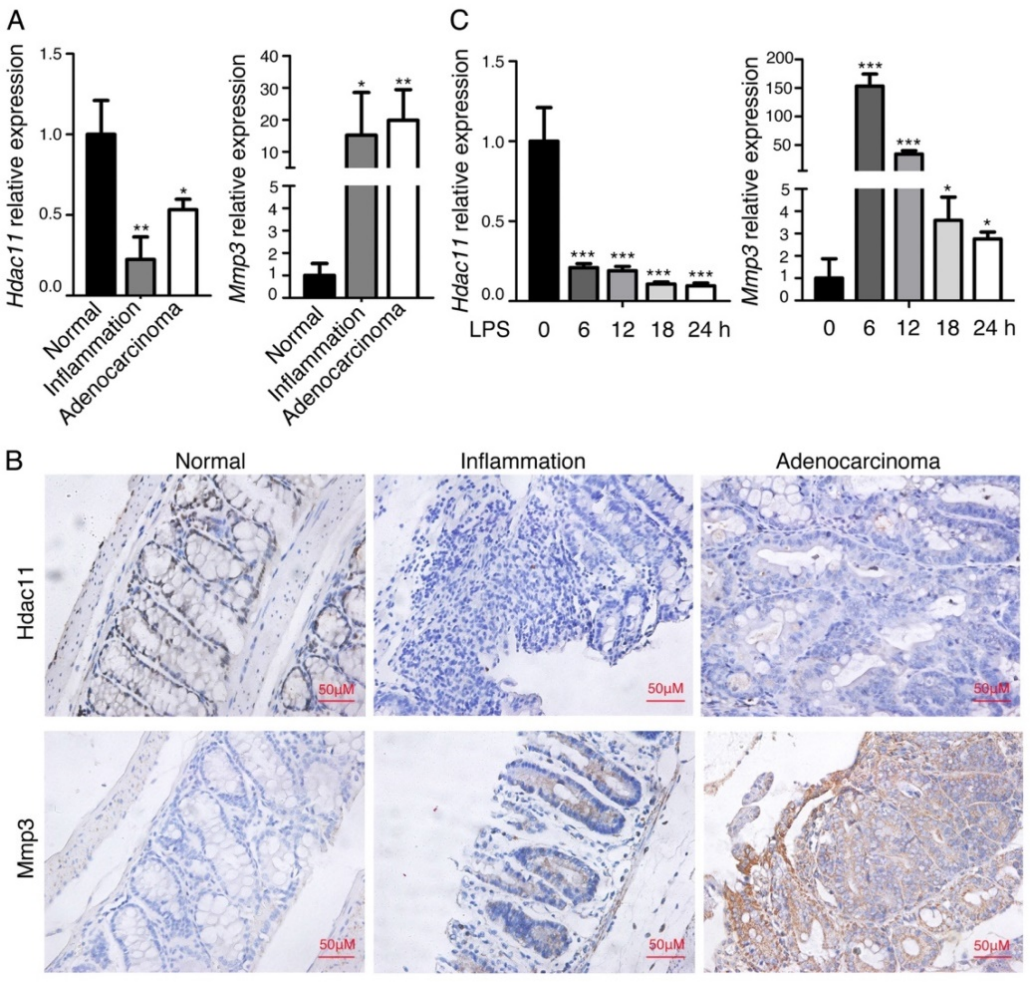
Hdac11 is downregulated and Mmp3 is upregulated in chemically-induced CAC mouse model. (A) The expressions of *Hdac11* and *Mmp3* in mouse colitis tissue, CAC tissue, and normal colon tissue was evaluated with qRT-PCR analysis. (B) Representative images of Hdac11 and Mmp3 expression in CAC mouse model using immunohistochemistry. Left panel indicates mouse normal colon tissue, middle panel indicates colitis tissue and right panel indicates adenocarcinoma tissue. (C) Kinetics of LPS-induced *Hdac11* and *Mmp3* expression in murine macrophages RAW264.7 cells. Error bars represent SD. *p < 0.05, **p < 0.01, ***p < 0.001 compared with control.

We previously analyzed the global gene expression profiles of murine CAC tissues, including inflamed lesions, dysplasia and carcinoma 15, and discovered that a panel of genes related to inflammation and carcinogenesis are dysregulated in the process from colitis to colorectal cancer. Among them, the member of histone deacetylase (HDAC), *Hdac11*, was significantly downregulated in the inflammation-cancer link, whereas, *Mmp3*, the member of matrix metalloproteinases, was dramatically upregulated. To confirm the finding, we further analyzed the mRNA expression levels of *Hdac11* and* Mmp3* in the same mouse model (including colitis, CAC and normal colon tissues). As shown in Fig. 1A, *Hdac11* was dramatically decreased in the “inflammation-adenocarcinoma” tissues compared with the expression level in normal colorectal tissue, on the contrary, expression level of *Mmp3* was increased. The protein levels of Hdac11 and Mmp3 were investigated by immunohistochemistry in normal, inflammation and adenocarcinoma colon tissues of the CAC mouse model (Fig. 1B), which was consistent with the qRT-PCR result. To further verifying our findings, we used lipopolysaccharide (LPS) to stimulate inflammation in murine macrophages (RAW264.7), and then measured *Hdac11* and* Mmp3* expression levels. The mRNA levels of *Hdac11* were dramatically decreased, whereas *Mmp3* was increased (Fig. 1C), indicating that Hdac11 is downregulated and Mmp3 is upregulated in the inflammation-cancer link.

### HDAC11 is downregulated in human colorectal cancer tissues and the decreased HDAC11 level is correlated with advanced clinical stage, lymph node metastasis

**Figure 2:**
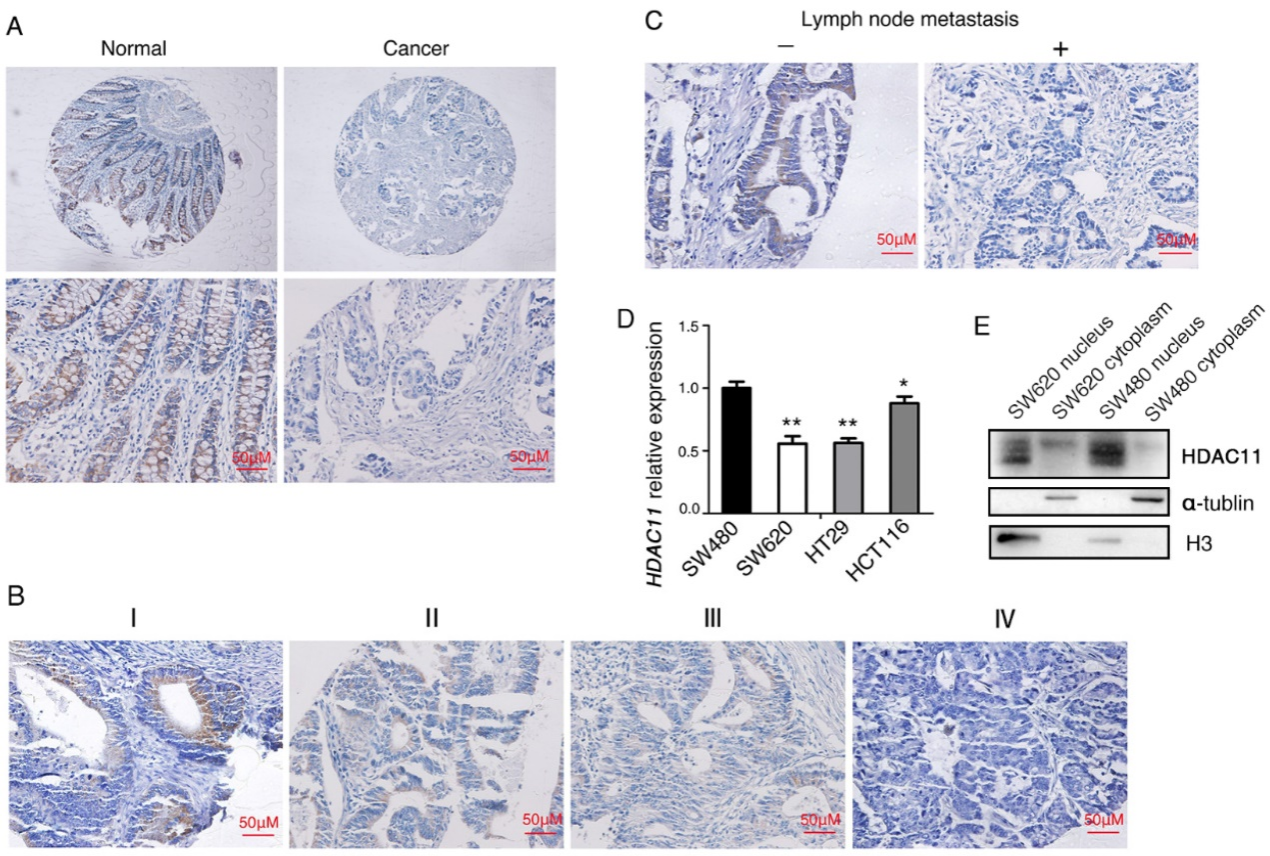
HDAC11 is downregulated and inversely correlated with lymph node metastasis in human colorectal cancer specimens. (A) Representative images of HDAC11 expression in human normal and colorectal cancer tissues. (B) Representative images of HDAC11 expression in different clinical stages. (C) Correlation between HDAC11 expression levels and lymph node (LN) metastasis status in carcinoma tissues. “-” indicates no metastasis”; “+” indicates with metastasis. (D) The mRNA expression of *HDAC11* in different invasive potential colon cancer cell lines tested by qRT-PCR. (E) The protein levels of HDAC11 in different invasive potential colon cancer cell lines tested by Western blotting. Error bars represent SD. *p < 0.05, **p < 0.01.

To generalize our findings, we evaluated the expressions of HDAC11 proteins in CRC tissues. Compared with normal tissues, HDAC11 protein is lowly expressed in CRC tissues and mainly localized in the nucleus of colorectal cells (Fig. 1B, Fig. 2A, Supplementary Fig. 1). Clinical samples were divided into two groups: low- HDAC11 expression (the HDAC11 expression score is less than 2) and high HDAC11 expression (the HDAC11 expression score is greater than 2). Expression levels of HDAC11 proteins were significantly lower in CRC tissues (30/103 cases, 29.1%) comparing to the distant normal mucosa (51/103 cases, 49.5%, p<0.01) (see Table 1). Based on the above results, we speculated that HDAC11 might act as a tumor suppressor in colorectal cancer.

Then we analyzed the potential clinicopathologic implications of altered HDAC11 expression. In the 103 individuals with colorectal carcinoma, the HDAC11 level inversely correlated with clinical stage (Fig. 2B), and lymph node metastasis (Fig. 2C) (P=0.029 and 0.029, respectively; Table 1). However, its level didn't correlate with age, or gender, or cell differentiation. Moreover, qRT-PCR analysis was used to verify *HDAC11* expression levels within the colon cancer cell lines. *HDAC11* mRNA levels were much lower in relatively high metastatic potential cell lines (SW620, HCT116 and HT-29), comparing to SW480 cell line (low metastatic potential cell line) (Fig. 2D). We extracted the cellular proteins of colorectal cancer cell lines SW620 and SW480, and detected the expression of HDAC11 by Western blotting. HDAC11 proteins were mainly expressed in the nucleus of the two cell lines, and the expression levels of HDAC11 were higher in SW480 (Fig. 2E). It suggested that the expression levels of HDAC11 might be related to metastatic potential of colon cancer cells. Above results indicated that HDAC11 could play pivotal roles in the development of colorectal cancer.

### Hdac11 down-regulates the expression level of Mmp3 partly through reducing the level of histone H3K9 acetylation at the promoter of *Mmp3*

**Figure 3:**
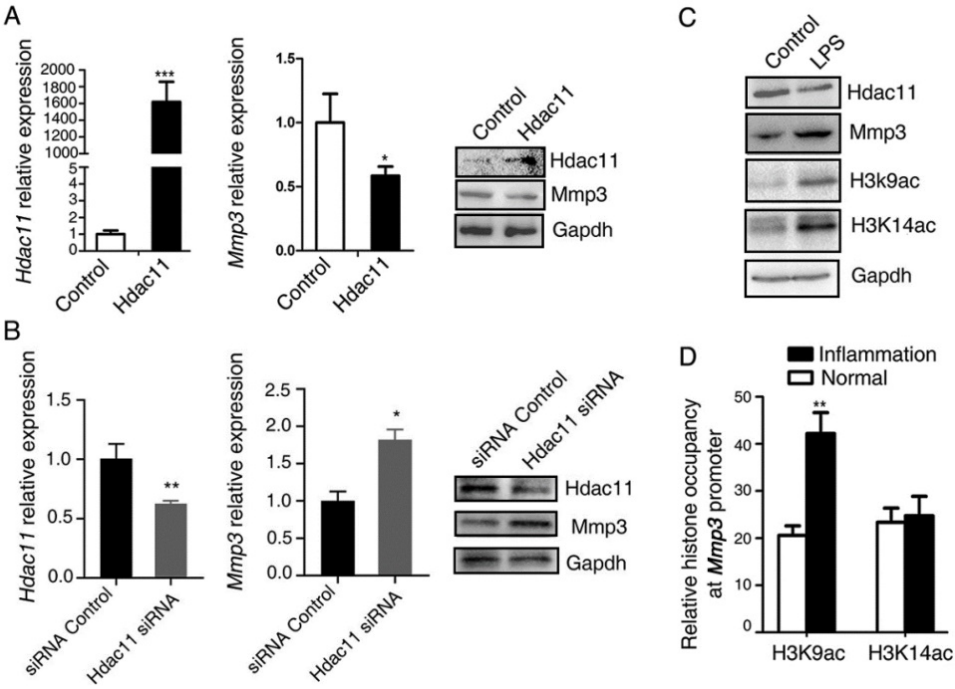
Hdac11 down-regulates the expression level of Mmp3 partly through reducing the level of histone H3K9 acetylation at the promoter of *Mmp3*. (A) PIRESneo3-HDAC11 was transfected into CT26.WT cells and an empty vector was served as a negative control. The expression of Hdac11 and Mmp3 was tested by qRT-PCR and Western blotting. (B) CT26.WT cells were transfected with siRNA targeting Hdac11 or control siRNA and the expression of Mmp3 was determined by qRT-PCR and Western blotting. (C) The murine macrophages (RAW264.7) were treated with LPS to induce an acute inflammation response, and the indicated proteins are assayed by Western blotting. (D) Comparing the histone occupancy in the *Mmp3* promoter between normal and inflammation tissues. Native ChIP assay was used to assay the levels of histone H3K9 and H3K14 acetylation at the *Mmp3* promoter in normal and inflammation colon tissues from the AOM/DSS-induced colitis mice model. Error bars represent SD.*p < 0.05, **p < 0.01, ***p < 0.001 compared with control.

Multiple studies have shown that MMP3 is pivotal for the invasion of many solid tumors, for instance, gastric cancer 16, ovarian cancer 17. In addition, the above results demonstrated that the expression levels of Mmp3 and Hdac11 have a negative correlation in the CAC mouse model. To test whether Hdac11 regulates the expression of Mmp3, we examined the effects of Hdac11 on Mmp3 expression. In CT26.WT cells, the mRNA and protein level of Mmp3 was decreased in response to Hdac11 overexpression (Fig. 3A). On the contrary, suppression of Hdac11 significantly upregulated the mRNA and protein levels of Mmp3 (Fig. 3B).

The major function of HDACs is to catalyze the removal of acetyl groups from the N-acetyllysines on histone and non-histone proteins, thus modify the chromatin structure and play pivotal roles in modulating gene transcription 18. The high level of H3K9 and H3K14 acetylation results in the gene transcriptional activation 19. In addition, recent studies reported that acute ethanol decreased the expression of HDAC11 and it is partly responsible for the mechanisms of the ethanol-induced gene expression through generating a global increase in H3K9ac and H3K14ac at these genes promoter 20. Thus, we questioned whether HDAC11 down-regulated the expressions of Mmp3 through reducing the levels of histone H3K9 and H3K14 acetylation at the promoter of *Mmp3*. As shown in Fig.3C, in the LPS-induced acute inflammation model, LPS downregulated the protein levels of Hdac11, whereas upregulated the levels of Mmp3, H3k9ac, and H3k14ac (Fig. 3C). We compared the histone occupancy in the Mmp3 promoter between normal and inflammation tissues. Native ChIP assay was used to assay the levels of histone H3K9 and H3K14 acetylation at the *Mmp3* promoter in normal and inflammation colon tissues from the AOM/DSS-induced colitis mice model. We revealed that histone H3K9 acetylation at *Mmp3* promoter was increasing, however, histone H3K14 acetylation at Mmp3 promoter had no difference in normal and inflammation tissues (Fig. 3D). This discovery suggested that under the inflammation circumstance, Hdac11 down-regulated the expression level of *Mmp3* may through reducing the level of histone H3K9 acetylation at the promoter of *Mmp3* gene, at least partly.

### HDAC11 inhibits colorectal cancer cell migration and invasion by down-regulating Mmp3 expression

**Figure 4:**
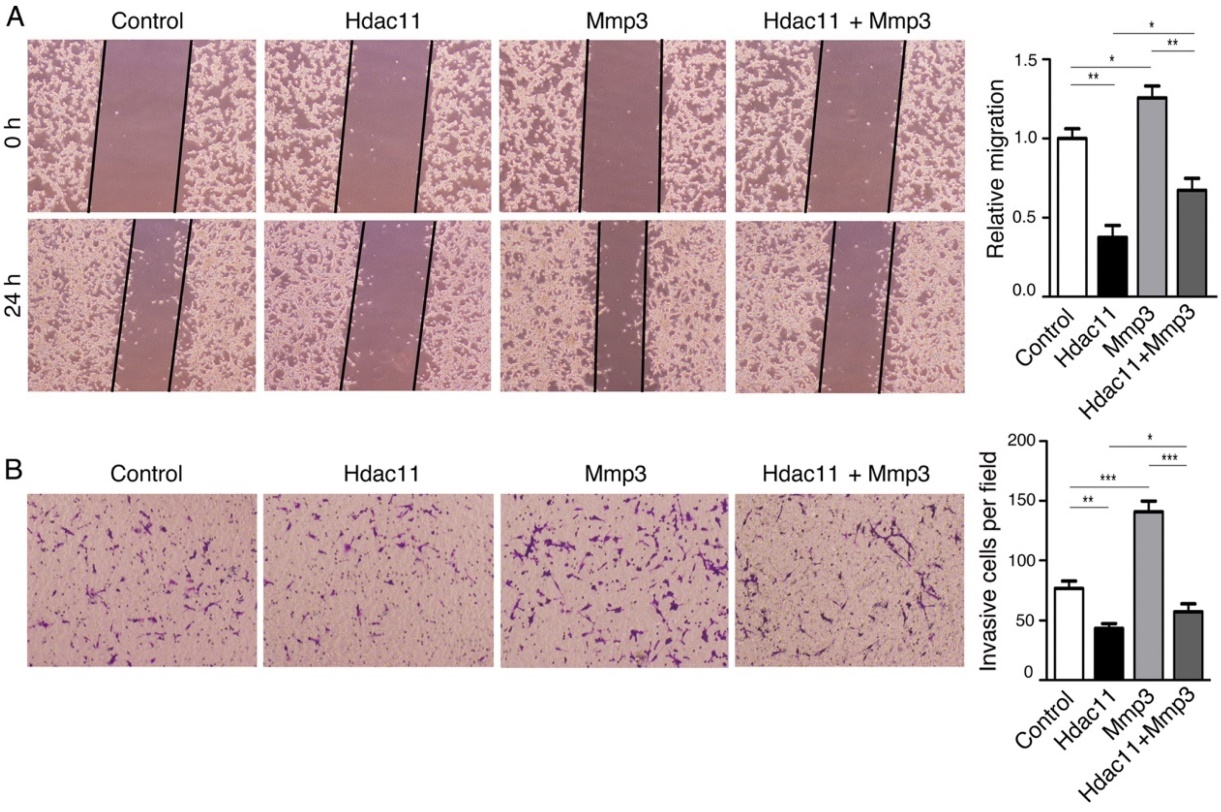
Hdac11 inhibits colorectal cancer cell migration and invasion by down-regulating Mmp3 expression. (A) Wound-healing assay indicated that Hdac11 suppressed the migration of CT26.WT cells. The weakened migration capabilities of colorectal cancer cells overexpressing Hdac11 could be rescued by transfecting with Mmp3 vector. (B) Transwell migration assay suggested that Hdac11 decreased the invasion ability of CT26.WT cells. The weakened invasion abilities of colorectal cancer cells overexpressing Hdac11 could be rescued by transfecting with Mmp3 vector. The figures show the representative images of the migrated stained cells. Cell numbers were counted in five randomly selected areas. Error bars represent SD. *p < 0.05, **p < 0.01, ***p < 0.001 compared with control.

The negative correlation between HDAC11 and lymph node metastasis that we found in colorectal cancer clinical specimens impelled us to explore whether HDAC11 plays a role in regulation of malignant biological behaviors of colorectal cancer cells, including migration, invasion and metastasis. Accordingly, we transiently transfected mouse CT26.WT colorectal cancer cells, which have high invasive and metastatic potential, with plasmids containing *Hdac11* cDNA and siRNAs targeting *Hdac11* (Fig. 3A, 3B). Next, we investigated whether changes in Hdac11 expression could affect the cell migration and invasion abilities in CT26.WT cells. Re-expression of Hdac11 markedly inhibited the CT26.WT cells' capability for migration (Fig. 4A) and invasion (Fig. 4B), whereas suppression of Hdac11 promoted the CT26.WT cells' capability for migration and invasion (Supplementary Fig. 2).

To explore whether the alteration of Mmp3 expression plays a critical role in HDAC11- mediated inhibition of colorectal cancer cell migration and invasion, we designed vectors encoding mouse Mmp3 and reintroduced it into CT26.WT cells. The results of the wound-healing assay and transwell assay demonstrated that HDAC11-meidated inhibition of CT26.WT cell migration and invasion was weakened in part by the re-expression of *Mmp3* (Fig. 4A, 4B). Thus, the results demonstrated that Hdac11 suppresses CT26.WT cells migration and invasion through inhibition of Mmp3 expression, at least partly.

## Discussion

The accumulation of genetic and epigenetic alterations plays pivotal roles in the oncogenesis and development of tumors 21, 22. Studies of the molecular mechanism of epigenetics have largely focused on mechanisms such as DNA methylation, noncoding RNAs and chromatin modifications 23, 24. These modifications include histone acetylation, methylation, ubiquitination, sumoylation and phosphorylation 25, 26. Histone acetylation is attributed to the combined effects of histone acetyltransferase (HAT) and histone deacetylation (HDAC). As is known to us, many members of the HDAC family play a critical role in promoting carcinogenesis. As reported previously, acute myeloid leukemia patients with lower HDAC1 level had better prognosis and HDAC1 may become a new molecular marker and target for clinical diagnosis, prognosis, and treatment of myeloid leukemia 27. Class II HDACs can promote growth of human hepatocellular carcinoma by induction of apoptosis and cell cycle arrest and play a role in pancreatic adenocarcinoma progression 28, 29. Therefore, HDACs become potential targets for anticancer therapeutics. Histone deacetylases (HDACs) have attracted a great interest as anticancer drug targets, and many HDAC inhibitors (HDACIs) have displayed clinical efficacy in treating specific tumors 30. Nevertheless, HDACs may inhibit tumorigenesis and progression in some neoplastic disease. As shown in the following examples, strong class I HDAC expression has been suggested as a positive survival prognosticator in breast cancer 31. Class II HDACs, in contrast, tended to be downregulated in human tumors and high expression in some tumors such as non-small cell lung carcinoma was linked to a better prognosis 32, 33. Histone deacetylase (HDAC) 10 can suppress cervical cancer metastasis through inhibition of Matrix Metalloproteinase (MMP) 2 and 9 expression 34. The past years has witnessed that therapeutic efficacy of some HDAC inhibitors in the treatment of tumors is limited. The reason may be that the function of each specific HDAC in tumorigenesis and progression is not fully understood. Therefore, dissecting the role of each HDAC in specific cancer has far-reaching significance.

HDAC11 is the most recently identified zinc-dependent HDAC family member and the only member of class IV HDACs. HDAC11 is highly conserved among different species, and in humans, its transcripts are highly expressed in kidney, heart, brain, skeletal muscle, and testis 8. Enforced expression of HDAC11 in HEK-293 cells followed by coimmunoprecipitation indicates that HDAC11 and HDAC6 interact directly or indirectly 8. Up to now, HDAC11 has been a rising star in epigenetics. In particular, its roles in carcinogenesis and tumor progression have attracted a substantial amount of attention. While the evidence accumulated to date provide evidence that HDAC11 may have many faces in different tumors. For example, some studies show that HDAC11 is overexpressed in hepatocellular carcinoma and renal pelvis urothelial carcinoma 35, 36, whereas it was downregulated in glioblastoma 37. However, few researches focused on the actual expression patterns of HDAC11 in colorectal cancer. In the current study, we found that HDAC11 expression level is lower in mouse colitis tissues and CAC tissue than in normal colon tissue. In addition, clinically HDAC11 expression is obviously downregulated in colorectal cancer specimens with lymph node metastasis. And HDAC11 is downregulated in the relative high metastatic potential colorectal cancer cells compared to the relative low metastatic potential colorectal cancer cells. Our data indicate that HDAC11 may be a metastasis suppressor in colorectal cancer. To our knowledge, we have described previously unknown and actual expression patterns of HDAC11 in carcinoma.

HDAC11 was recently shown to regulate many genes expression. For example, HDAC11 gene silencing increased PAI-1 expression, whose inhibition protected the kidney from I/R-induced inflammation and functional loss 38. Previously, Alejandro Villagra et al. 9 had identified HDAC11 as a negative transcriptional regulator of Il10 expression in mouse and human APCs. Moreover, HDAC11 was shown to play an essential role in regulating OX40L expression 39. In this study, we indicated that HDAC11 down-regulated the expression level of Mmp3 partly through reducing the level of histone H3K9 acetylation at the promoter of Mmp3. Nevertheless, the function of HDAC11 remains poorly understood particularly in genesis and progression of cancer. In our system, HDAC11 overexpression had a remarkable impact on migration and invasion abilities of colorectal cancer cells. The metastasis suppression functions of HDAC11 are depended on regulating MMP3 expression, because re-expressing MMP3 CDS could reverse HDAC11's effect. In summary, our study shows that HDAC11 inhibits tumor metastasis in CRC through suppressing MMP3 expression.

Our observation and findings in this study demonstrate several novel features. Firstly, our data underline the actual expression pattern of HDAC11 in colorectal cancer. Secondly, these studies also provide important insights into the role of HDAC11 in colorectal carcinoma invasion and metastasis. Furthermore, to our knowledge, this study provides the first regulation links between HDAC11 and MMP3. In conclusion, our data suggest HDAC11 as a potential novel target for controlling the invasion and metastasis of colorectal cancer.

## Supplementary Material

Supplementary figures.

## Figures and Tables

**Table 1:** Association between HDAC11 expression and clinicopathological features from colorectal cancer patients (n=103)

Parameter	n=103	Expression level		*P* value
		Low N (%)	High N (%)	
**Histologic type**				
Non-cancer tissues	103	52(50.5%)	51(49.5%)	0.003
Cancer tissues	103	73(70.9%)	30(29.1%)	
**Age (years)**				
≤56	55	43(78.2%)	12(21.8%)	0.081
>56	48	30(62.5%)	18(37.5%)	
**Gender**				
Male	58	39(67.2%)	19(32.8%)	0.357
Female	45	34(75.6%)	11(24.4%)	
**Lymph node metastasis**				
Absent	48	29(60.4%)	19(39.6%)	0.029
Present	55	44(80.0%)	11(20.0%)	
**Differentiation grade**				
High/ Moderate	88	59(67.0%)	29(33.0%)	0.078
Low	15	14(93.3%)	1(6.7%)	
**TNM stage**				
Ⅰ-Ⅱ	48	29(60.4%)	19(39.6%)	0.029
Ⅲ-Ⅵ	55	44(80.0)	11(20.0%)	
