# Supplementary material for: Histone deacetylase (HDAC) 11 inhibits matrix metalloproteinase (MMP) 3 expression to suppress colorectal cancer metastasis
Source: J Cancer. 2022 Mar 28;13(6):1923–32. doi: 10.7150/jca.66914 (PMC8990422; doi:10.7150/jca.66914)
Supplement: Supplementary file 1 — Supplementary figures. [file jcav13p1923s1.pdf]

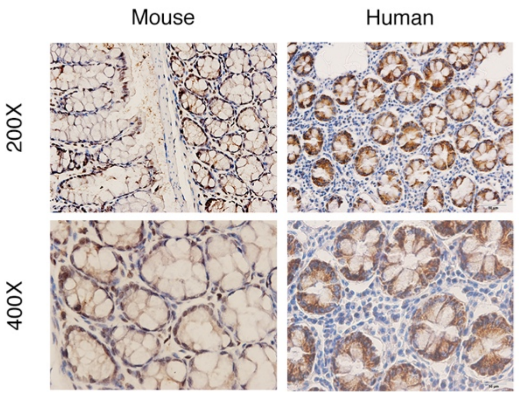


**Supplementary Figure 1.** Most of the HDAC11 proteins are located in the nucleus of normal colon tissue. Representative images of HDAC11 expression in mouse and human normal tissues. Bar represents 50 μm.

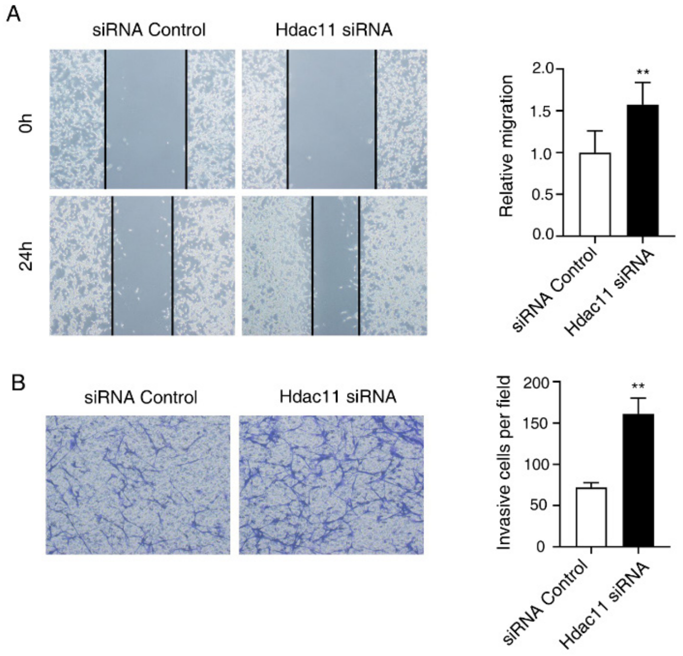


**Supplementary Figure 2.** Suppression of HDAC11 markedly promoted the migration and invasion capabilities of colon cancer cells CT26.WT. (A) Wound-healing assay indicated that the suppression of HDAC11 promoted the migration abilities of CT26.WT cells. (B) Transwell assay indicated that the knockdown of HDAC11 increased the invasion ability of CT26.WT cells. The figures show the representative images of the migrated stained cells. Cell numbers were counted in five randomly selected areas. Error bars represent SD. **p < 0.01 compared with control.
